# The TyrR Transcription Factor Regulates the Divergent *akr-ipdC* Operons of *Enterobacter cloacae* UW5

**DOI:** 10.1371/journal.pone.0121241

**Published:** 2015-03-26

**Authors:** Thomas J. D. Coulson, Cheryl L. Patten

**Affiliations:** Department of Biology, University of New Brunswick, Fredericton, Canada; Belgian Nuclear Research Centre SCK•CEN, BELGIUM

## Abstract

The TyrR transcription factor regulates genes involved in the uptake and biosynthesis of aromatic amino acids in *Enterobacteriaceae*. Genes may be positively or negatively regulated depending on the presence or absence of each aromatic amino acid, all three of which function as cofactors for TyrR. In this report we detail the transcriptional control of two divergently transcribed genes, *akr* and *ipdC*, by TyrR, elucidated by promoter fusion expression assays and electrophoretic mobility shift assays to assess protein-DNA interactions. Expression of both genes was shown to be controlled by TyrR via interactions with two TyrR boxes located within the *akr-ipdC* intergenic region. Expression of *ipdC* required TyrR bound to the proximal strong box, and is strongly induced by phenylalanine, and to a lesser extent by tryptophan and tyrosine. Down-regulation of *akr* was reliant on interactions with the weak box, and may also require a second, as yet unidentified protein for further repression. Tyrosine enhanced repression of *akr*. Electrophoretic mobility shift assays demonstrated that TyrR interacts with both the strong and weak boxes, and that binding of the weak box *in vitro* requires an intact adjacent strong box. While the strong box shows a high degree of conservation with the TyrR binding site consensus sequence, the weak box has atypical spacing of the two half sites comprising the palindromic arms. Site-directed mutagenesis demonstrated sequence-specific interaction between TyrR and the weak box. This is the first report of TyrR-controlled expression of two divergent protein-coding genes, transcribed from independent promoters. Moreover, the identification of a predicted aldo-keto reductase as a member of the TyrR regulon further extends the function of the TyrR regulon.

## Introduction

The TyrR transcription factor controls the expression of genes involved in the uptake and metabolism of aromatic amino acids. In *Escherichia coli* K12, the TyrR regulon consists of eight operons and nine genes [1, 2]. Regulation of each transcriptional unit is either positive or negative, depending on the position of TyrR binding sites, known as TyrR boxes, in the promoter region. TyrR boxes that share ten or more nucleotide matches with the consensus sequence (TGTAAA-N_6_-TTTACA) are defined as strong, while those with fewer then ten matches are referred to as weak boxes [3]. An invariant feature of known and functional TyrR boxes are the G-C residues of each palindromic arm, spaced 14 base pairs apart, which are essential for TyrR binding [4]. Strong boxes are bound by TyrR with high affinity *in vivo*, whereas weak boxes require an adjacent strong box to bind TyrR [5]. The three aromatic amino acids tryptophan, phenylalanine and tyrosine function as cofactors for TyrR, interacting with the protein dimer at two distinct sites and increasing its affinity for binding DNA [1, 6]. In the cytosol, TyrR exists as a homo-dimer, comprised of two 57 kDa subunits. However, in the presence of tyrosine and ATP, TyrR undergoes further oligomerization to form a hexamer [7], allowing it to interact with multiple DNA binding sites [8].

TyrR typically represses transcription by preventing access of the RNA holoenzyme to the core promoter, or inhibiting the later stages of open complex formation and elongation. A strong TyrR box overlapping the −35 element represses expression of the *E*. *coli tyrP* gene in the presence of tyrosine [4, 9]. A similarly positioned strong TyrR box, whose sequence perfectly matches the consensus, represses *aroG* in the presence of phenylalanine and tryptophan [10]. TyrR auto-regulates its own expression, repressing the *tyrR* gene by binding upstream of the −35 sequence [11, 12]. TyrR binding sites downstream of the core promoter may also repress transcription by occluding the transcription start site, as is the case for *tyrB* in the presence of tyrosine [9]. Expression of the *aroP* gene from its primary promoter P1 is inhibited when TyrR, bound to two sites downstream of the *aroP* transcription start site, recruits RNA polymerase to an alternate promoter P3 which overlaps the P1 promoter but is located on the opposite DNA strand. This recruitment prevents open complex formation at P1, thereby inhibiting transcription [13–15]. Repression of the *mtr* promoter by TyrR is also dependent on DNA structure; negative supercoiling stabilized by the DNA binding proteins IHF and HU is necessary for repression with tryptophan [16].

While the primary mechanism of TyrR transcriptional control is repression, the expression of several genes is enhanced by TyrR. The *mtr* gene of *E*. *coli* contains a pair of adjacent TyrR boxes, one strong (proximal to the promoter) and one weak (distal to the promoter), upstream of the −35 sequence, and separated from each other by eight base pairs [17, 18]. In the presence of tyrosine, TyrR binds to the strong box only, thereby activating transcription of *mtr*. In the presence of phenylalanine, TyrR binds to both sites and further enhances *mtr* transcription above the level of induction by tyrosine [17, 19, 20]. *folA* is activated via hexameric TyrR in response to tyrosine. Binding of IHF within the *folA* promoter induces DNA bending and facilitates TyrR interaction with RNA polymerase to enhance transcription [2]. Up-regulation of genes by TyrR is dependent on interactions between residues in the N-terminal domain of TyrR and the alpha subunit C-terminal domain (αCTD) of RNA polymerase [21].

In *E*. *coli*, experimental evidence has shown that the TyrR regulon is restricted to genes required for aromatic amino acid uptake and biosynthesis. However, bioinformatic and functional analysis suggests that TyrR homologues in other bacteria have expanded or different regulons. Microarray assays indicated that the TyrR homologue in pseudomonads, PhhR, is a much more global regulator, controlling 21 genes which, in addition to proteins for aromatic amino acid uptake and biosynthesis, encode enzymes for catabolism of phenylalanine and other aromatic compounds, ABC transporters, a LysR type transcription factor, and several proteins of unknown function [22, 23]. Using sequence analysis and comparative genomics to identify transcription networks of *Shewanella* spp., the orthologous TyrR was predicted to control a regulon that comprises 35 genes spanning 16 operons [24]. The *Shewanella* TyrR orthologue has undergone regulon expansion, and is proposed to regulate expression of genes for branched chain and aromatic amino acid degradation and components of the glyoxylate shunt of the tricarboxylic acid cycle [24].

**Fig 1:**
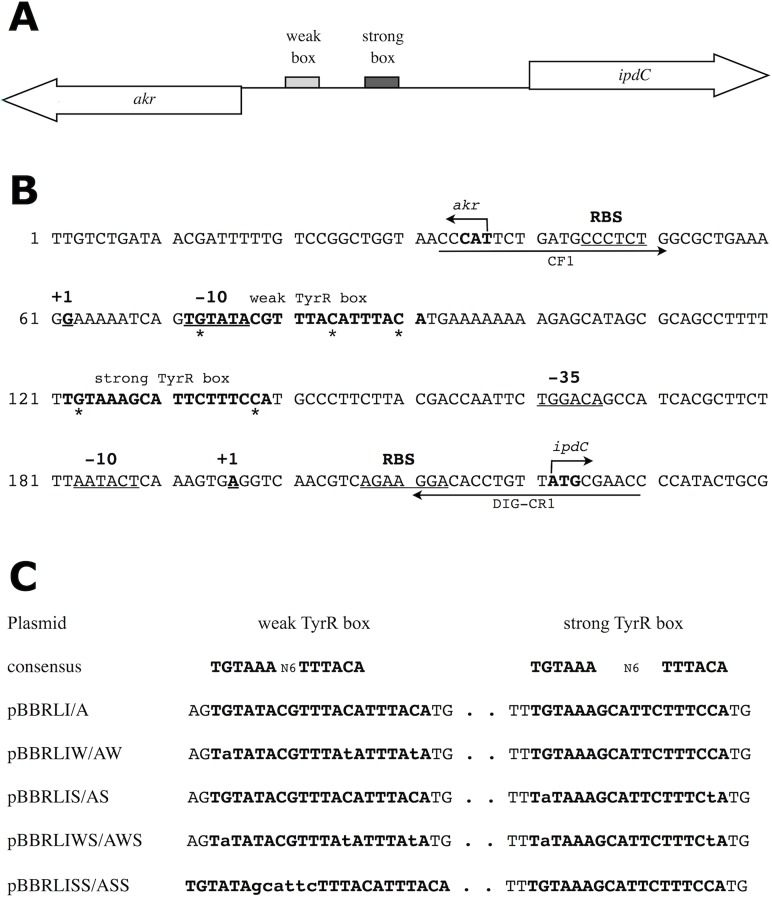
The *akr-ipdC* regulatory region of *E*. *cloacae* UW5. (A) Relative location of the two TyrR binding sites located between the *akr* and *ipdC* genes. (B) Nucleotide sequence of the *akr-ipdC* intergenic region. TyrR boxes are in bold and nucleotides proposed to be essential are indicated with asterisks. The predicted −10, −35 elements and ribosome binding sites for both genes are underlined. The identified *ipdC* transcription start site, and predicted *akr* transcription start site are in bold underline. Arrows above the sequence indicate the *akr* and *ipdC* start codons. Arrows below the sequence indicate primer binding sites for CF1 and DIG-CR2. (C) Reporter gene expression plasmids driven by the *ipdC* (I) or *akr* (A) promoters, with the corresponding mutation or insertions indicated in lowercase compared to the TyrR box consensus sequence.

Previously, we showed that in the rhizobacterium *Enterobacter cloacae* UW5, the TyrR regulon includes the *ipdC* gene encoding the enzyme indolepyruvate decarboxylase which converts indole-3-pyruvate to indole-3-acetaldehyde, a key step in the indole-3-acetic acid (IAA) biosynthetic pathway [25]. Binding of TyrR to a sequence with high similarity to the consensus binding sequence is required for activation of *ipdC* expression and IAA production. This strong TyrR box is centered directly between *ipdC* and a divergently transcribed gene, *akr*, encoding a putative aldo-keto reductase (Fig. 1A). In addition, a second sequence that resembles a TyrR box was found upstream of the *akr* coding sequence (Fig. 1A and B). This second site contains palindromic arms with high identity to those of the consensus TyrR binding sequence but with non-canonical spacing between them (hereafter referred to as the weak TyrR box). In this study, we determined that *akr* is a member of the TyrR regulon and that TyrR binds to both predicted sites in the *akr-ipdC* intergenic region. Using reporter gene expression assays, site-directed mutagenesis and electrophoretic mobility shift assays (EMSAs), we characterize the roles of the strong and weak TyrR boxes in the activation and repression of *ipdC* and *akr*, respectively. Genes that are co-regulated are often functionally related and understanding how *akr* is controlled by TyrR is a first step towards elucidating its possible role in production of IAA, an important signaling molecule in bacterial-plant interactions [26].

## Materials and Methods

### Bacterial strains and culture conditions

The bacterial strains and plasmids used in this study are listed in Table 1. *E*. *coli* and *E*. *cloacae* were routinely grown at 37°C and 30°C, respectively, in either Luria Bertani (LB) broth or M9 minimal media (Difco). Media was supplemented with antibiotics when required at final concentrations of 100 μg/mL ampicillin, 25 μg/mL kanamycin, 20 μg/mL gentamicin or 5 μg/mL tetracycline. L-Aromatic amino acid supplements to the growth media were supplied at a final concentration of 1 mM when indicated.

**Table 1:** Bacterial strains and plasmids used in this study.

Strains or Plasmids		Relevant characteristics	Reference
*E*. *coli*			
	JM109	Cloning host; *endA1 recA1 gyrA96 thiA hsdR17 relA1 supE44 Δlac*-*proAB* F’ *traD36 proAB laqI*q *Δlac*Z M15	[27]
	S17-λ pir	Cloning host; *pir recA thi pro hsdR* M RP4:2 Tc:Mu:Km Tn*7* Tpr, Str^s^	[28]
	M15	Expression host; *E*. *coli* K12 *ΔlacZ Δmtl* pREP4, Str^s^	Qiagen
*E*. *cloacae*			
	UW5	Wild-type strain	[29]
	J35	UW5 *tyrR*::*Tc* ^r^	[25]
Plasmids			
	pGEM-T Easy	Cloning vector, Amp^r^	Promega
	pGEM-IP1	420 bp 5’-RACE *ipdC* fragment cloned into pGEM-T Easy, Amp^r^	This study
	pGEM-AP1	280 bp 5’-RACE *akr* fragment cloned into pGEM-T Easy, Amp^r^	This study
	pQEtyrR	1542 bp fragment encoding the *E*. *cloacae* UW5 *tyrR* gene cloned into pQE-30, Amp^r^	[25]
	pJQSIPG	pJQ200SK derivative; P*ipdC*::*uidA*, Gm^r^	[25]
	pJQSIAPG	pJQ200SK derivative; P*akr*::*uidA*, Gm^r^	[25]
	pBBR1MCS-2	Cloning vector, Km^r^	[28]
	pBBRL	pBBR1MCS-2, P*lacZ* deletion, Km^r^	This study
	pBBRLI	2.3 kb P*ipdC*::*uidA* fragment from pJQSIPG cloned into pBBRL, Km^r^	This study
	pBBRLIW	pBBRLI derivative, mutation of the TyrR weak box, Km^r^	This study
	pBBRLIS	pBBRLI, mutated strong box, Km^r^	This study
	pBBRLIWS	pBBRLLI, mutated weak and strong box, Km^r^	This study
	pBBRLISS	pBBRLI, weak to strong box, Km^r^	This study
	pBBRLA	2.3 kb P*akr*::*uidA* fragment from pJQSIAPG cloned into pBBRL, Km^r^	This study
	pBBRLAW	pBBRLA, mutated weak box, Km^r^	This study
	pBBRLAS	pBBRLA, mutated strong box, Km^r^	This study
	pBBRLAWS	pBBRLA, mutated weak and strong box, Km^r^	This study
	pBBRLASS	pBBRLA, weak to strong box, Km^r^	This study

### Transcription start site identification

5’-Rapid amplification of cDNA ends (RACE) was used to identify the transcription start sites of *akr* and *ipdC*. Overnight cultures of *E*. *cloacae* UW5 were inoculated 1:100 into fresh M9 media with or without 1 mM of each aromatic amino acid (L-tryptophan, L-phenylalanine, L-tyrosine). Cultures were grown to mid-logarithmic (OD_600_ = 0.6, 8 h) and stationary (OD_600_ = 2.5, 48 h) phase, at which point 1 mL of culture was transferred to a microfuge tube containing 0.5 mL RNAprotect (Qiagen), and vortexed for ~30 s. Cells were then centrifuged, supernatant discarded and the pellet stored at −20°C. Total bacterial RNA was isolated using the RNeasy Mini Kit (Qiagen). RNA quality was assessed by running a sample on a 2% agarose gel, and RNA quantity and purity was measured spectrophotometrically at 260 nm using a SpectraMax M5 plate reader (Molecular Devices).

A total of 2 μg RNA was treated with amplification grade DNase I (Invitrogen) to remove any contaminating DNA. cDNA synthesis was performed using Superscript III reverse transcriptase (Invitrogen) according to the manufacturer’s recommendations with primers IPIF and IPDC-GSP1 (Table 2) for *akr* and *ipdC*, respectively. cDNA was purified using Qiagen’s PCR purification kit and end tailed with dCTP using terminal transferase (New England Biolabs) in a reaction containing 1X TdT buffer, 2.5 mM CoCl_2_ and 10 mM dCTP. Reactions were incubated at 95°C for two min and cooled to room temperature before addition of 20 U terminal transferase and further incubation at 37°C for 20 min. Tailing reactions were stopped by heating to 65°C for 10 min.

**Table 2:** PCR primers used in this study.

Primer	Sequence 5’-3’
IP1F	GCCATGGCAGGAAATCTTC
IPA2R	GACGGTCCAGCAGGTAATG
U2R	TTCCACAGTTTTCGCGATCC
AAP	GGCCACGCGTCGACTAGTACGGGIIGGGIIGGGIIG
AUAP	GGCCACGCGTCGACTAGTAC
AKR-GSP2	CGAAATTACGTTCGGCTGAG
IPDC-GSP1	ACATGCTCGGCATAGCTG
IPDC-GSP2	AACGCCGAATGTGGTCAG
CF1	CCCATTCTGATGCCCTCTG
CR1	GGTTCGCATAACAGGTGTCC
DIG-CR1	DIG-GGTTCGCATAACAGGTGTCC
WFT1	TGTATACGTTTACATTTACATGAAAAAAAAGAGCATAGCG
WFS1	TGAAAAAAAAGAGCATAGCGCAGCC
WRT1	TGTAAATGTAAACGTATACACTGATTTTTCCTTTCAGC
WRS1	CTGATTTTTCCTTTCAGCGCCAGAGG
WFT2	TATATACGTTTATATTTATAtgaaaaaaaagagcatagcgcagcc
WRT2	TATAAATATAAACGTATATActgatttttcctttcagcgccaga
SFT1	TATAAAGCATTCTTTCTATGCCCTTCTTACGACCAATT
SFS1	TGCCCTTCTTACGACCAATTCTGGA
SRT1	TAGAAAGAATGCTTTATAAAAAGGCTGCGCTATGC
SRS1	AAAAAGGCTGCGCTATGCTCTTTTTTTTCA

Ten microliters of tailed cDNA were then used in a 50 μL touchdown PCR with an abridged anchored primer (AAP) and gene specific primers, AKR-GSP2 and IPDC-GSP2 (Table 2) for *akr* and *ipdC*, respectively. Touchdown PCR cycling conditions were as follows: initial denaturation at 94°C for 2 min; 20 cycles of 94°C for 30 s, 58°C −0.5°C per cycle for 30 s, 72°C for 30 s; 25 cycles of 94°C for 30 s, 48°C for 30 s, 72°C for 30 s; final elongation at 72°C for 7 min. Ten microliters of PCR products were used as templates in a second round of PCR performed with a common AUAP primer (Table 2) and gene specific primers AKR-GSP2 or IPDC-GSP2 in 50 μL reaction volumes. Cycling conditions were as follows: initial denaturation at 94°C for 2 min; 35 cycles of 94°C for 30 s, 57°C for 30 s, 72°C for 30 s; final elongation at 72°C for 7 min. PCR products were gel purified before cloning.

Both *akr* and *ipdC* tailed cDNA fragments were ligated into pGEM-T Easy (Promega), generating the plasmids pGEM-IP1 and pGEM-AP1 (Table 1), respectively, and subsequently transformed into *E*. *coli* JM109 cells (Promega). Inserts were then sequenced using standard T7 and SP6 primers (Robarts Research Institute, London, Ontario, Canada) to identify the transcription start site.

### Construction of reporter gene fusions and promoter mutagenesis

The impact of the TyrR transcription factor on expression of *akr* and *ipdC* was examined using plasmid-based reporter gene fusions. The suicide plasmids pJQSIPG and pJQSIAPG contain the *ipdC* and *akr* promoters, respectively, fused to the *uidA* reporter gene encoding β-glucurionidase [25]. The P*ipdC*::*uidA* and P*akr*::*uidA* fusions were excised as ApaI-XbaI fragments from pJQSIAPG and pJQSIAPG, respectively, and inserted into a similarly digested derivative of the pBBR1MCS-2 broad host range plasmid [30] lacking the *lacZ* promoter (pBBRL). The *lacZ* promoter was excised from pBBR1MCS-2 using the restriction enzymes KpnI and NsiI. Remaining 5’ overhangs were removed by Klenow digestion followed by re-circularisation by blunt end ligation with T4 DNA ligase (NEB) to generate pBBRL. Following insertion of the P*ipdC*::*uidA* and P*akr*::*uidA* fusions, the resulting plasmids pBBRLI and pBBRLA, respectively, (Table 1) were transformed into *E*. *coli* JM109 (Promega), purified and introduced into *E cloacae* UW5 and J35 by electroporation.

The involvement of two predicted TyrR binding sites was examined by introducing point mutations into the binding sites using site-directed ligase-independent (SLIM) PCR mutagenesis [31] with the pBBRLI and pBBRLA plasmids as templates. SLIM-PCR utilises inverse PCR amplification of the template plasmid with two pairs of primers, a tailed pair containing the mutation and a short pair flanking the mutation site. Post-amplification denaturation and re-annealing produces a mixed population of products, of which only those containing the targeted mutation are capable of hybridization and therefore re-circularization. Mutations were generated in 25 μL PCR reactions using Phusion High Fidelity DNA polymerase (NEB) and 8% DMSO with the following sets of primer pairs: weak box mutagenesis, WFT2 and WRT2; WFS1 and WRS1; strong box mutagenesis, SFT1 and SRT1; SFS1 and SRS1; weak box conversion to strong box, WFT1 and WRT1; WFS1 and WRS1 (Table 2). A double TyrR box mutant was generated by introducing mutations in the wild-type strong box sequence of a previously generated weak box mutant plasmid. Cycling conditions for all SLIM-PCR reactions were as follows: 98°C for 3 min; 35 cycles of 98°C for 10 s; 63°C for 15 s; 72°C for 3 min; final elongation at 72°C for 5 min. Following amplification, 25 μL of PCR product was treated with 10 U DpnI at 37°C for 60 min with 5 μL D-buffer (20 mM MgCl_2_, 20 mM Tris-CL, pH 8.0, 5 mM DTT) to remove non-mutated template DNA. The reaction was stopped with 30 μL H-buffer (300 mM NaCl, 50 mM Tris-CL pH 9.0, 20 mM EDTA, pH 8.0) before SLIM-PCR hybridisation at 99°C for 3 min; followed by two cycles of 65°C for 5 min; 30°C for 40 min. Plasmid DNA was purified and transformed into CaCl_2_ competent *E*. *coli* S17-1 (λ*pir*) cells. Successful mutagenesis was confirmed by sequencing the plasmids (Robarts Research Institute). Following sequence confirmation, plasmids were introduced into wild-type *E cloacae* UW5 and *tyrR* null mutant *E*. *cloacae* J35 by electroporation. The resulting plasmids are listed in Table 1, with their corresponding mutations in Fig. 1C.

### Reporter gene expression assays

*ipdC* and *akr* promoter-driven reporter gene expression was quantified using the method of Cowie *et al*. (2006) [32]. Wild-type *E*. *cloacae* UW5 and *tyrR* null mutant *E*. *cloacae* J35 cells containing the reporter gene constructs described above were grown in triplicate from independent colonies overnight in LB broth with appropriate antibiotics, pelleted, and washed twice in 1X M9 salts. Cultures were then diluted 100-fold into 1 mL M9 minimal media in 2.2 mL 96-deep well plates (Eppendorf, Canada), with or without each of the aromatic amino acids. Cultures were grown to mid-logarithmic (OD_600_ = 0.6, 8 h) and stationary (OD_600_ = 2.5, 48 h) phase, at which time they were assayed for β-glucuronidase activity. Briefly, 20 μL of cell culture were withdrawn to wells of a 96-well plate (Cellstar, Sigma) containing 80 μL of reaction buffer (50 mM sodium phosphate [pH 7], 50 mM dithiothreitol, 1 mM EDTA, 0.0125% sodium dodecyl sulfate) and 0.44 mg/mL *p*-nitrophenyl β-D-glucuronide (PNPG), and incubated at room temperature until development of a strong yellow precipitate, at which time the reaction was stopped with the addition of 100 μL 1 M Na_2_CO_3_. Absorbance of the reaction products was measured at 405 nm using a SpectraMax M5 plate reader (Molecular Devices) and used to calculate specific promoter activity in Miller units (1000 x OD_405nm_)/(time x OD_600nm_ x culture volume in reaction). Wild-type *E*. *cloacae* UW5 and uninoculated medium were included as controls to assess background levels of absorbance at 405 nm.

### Electrophoretic mobility shift assays

The previously constructed expression vector pQEtyrR (Table 1) was used to purify TyrR via an N-terminal His_6_ tag [25]. *E*. *coli* M15 carrying plasmids pREP4, which constitutively produces high levels of *lac* repressor protein, and pQEtyrR were grown at 37°C in LB to mid logarithmic phase (OD_600_ = 0.6, 2.5 h) before induction with 1 mM isopropyl β-D-thiogalactopyranoside (IPTG) and allowed to incubate for an additional four hours. His_6_-TyrR was purified under native conditions using the QIAexpressionist Ni-NTA metal affinity chromatography kit (Qiagen). The native purification protocol was followed according to the manufacturer’s instructions with the exception of a modified wash step and elution buffer. Wash buffer 1 contained 50 mM NaH_2_PO_4_, 300 mM NaCl, 10 mM imidazole; wash buffer 2 was as described for wash buffer 1 with 10% glycerol; the elution buffer contained 50 mM NaH_2_PO_4_, 300 mM NaCl, 250 mM imidazole, 8% glycerol. Purified protein was quantified using the Quick Start Bradford protein assay (Bio-Rad), and purity assessed on a 12% SDS-PAGE gel stained with Coomassie brilliant blue G-250.

DNA fragments encompassing the intergenic region of *akr* and *ipdC* were amplified with Phusion High Fidelity DNA polymerase (NEB) using the wild-type and mutant promoter fusion plasmids described above as templates. Primers CF1 and DIG-CR1 (Table 2) amplified a 198 bp fragment spanning the full intergenic region (Fig. 1B), while incorporating a digoxigenin moiety (DIG) at one end. Non-DIG labeled primer CR1 was used in conjunction with CF1 to amplify unlabelled wild-type intergenic DNA to be used in competition assays. DNA fragments were separated on a 6% native-PAGE gel and stained with ethidium bromide before target bands were cut out and purified using a PCR purification kit (Qiagen).

Protein-DNA binding reactions were carried out as described previously [25]. Purified His_6_-TyrR was incubated at specified concentrations (87 nM, 438 nM, 877 nM, 1750 nM) with 1 μg poly d(I-C), 0.1 mM ATP and 0.1 mM of either L-tryptophan, L-phenylalanine or L-tyrosine in binding buffer (20% glycerol, 250 mM NaCl, 50 mM Tris-Cl [pH 7.5], 5 mM MgCl_2_, 2.5 mM dithiothreitol, 2.5 mM EDTA) for 5 min at room temperature. Following incubation, 50 ng of specific DIG-labeled DNA was added and incubated at room temperature for a further 30 min. Competition assays were performed by incubating His_6_-TyrR first with 100-fold molar excess unlabelled wild-type DNA (500 ng) for 5 min in reaction buffer at room temperature, followed by addition of 50 ng wild-type DIG-labeled DNA. Binding reactions were stopped with 5 μL loading buffer (0.25x Tris-Borate-EDTA, 60% glycerol, 0.02% bromophenyl blue), and loaded onto a 6% native-PAGE gel (6% acrylamide:bis-acrylamide [37:1], 2.5% glycerol, 0.5X Tris-borate-EDTA, 0.1 mM ATP, 0.1 mM either L-tryptophan, L-phenylalanine or L-tyrosine, 0.15% ammonium persulfate, 0.1% TEMED). Protein-DNA complexes were resolved at 80 volts for 2–3 h at 4°C.

DNA was transferred to a positively charged nylon membrane (Roche) using a Trans-Blot SD Semi-Dry cell (Bio-Rad) and visualised using the anti-DIG-Detection system according to the manufacturer’s instructions (Roche). Developed membranes were imaged with a Bio-Rad ChemiDoc MP (Bio-Rad).

### Statistical analysis

Statistically significant differences in gene expression from each promoter during logarithmic or stationary growth phases were detected by two-way analysis of variance (ANOVA) with post-hoc comparisons by TukeyHSD test using JMP 7 [33]. The genotype (i.e., strains UW5 and J35), promoter mutation, and amino acid treatments were treated as fixed effects, fully crossed with each other when indicated for each test. Statistical differences were defined as *p* values of <0.05. Unless stated otherwise, all main effects and interactions were significant.

## Results

### Mapping of the ***akr*** and ***ipdC*** promoters

To understand how TyrR regulates *akr* and *ipdC*, the location of the promoters of both genes relative to the predicted TyrR boxes was first determined. 5’-RACE was used to identify the transcription start sites from which the core promoter sequences of the two genes were inferred. The transcription start site for *ipdC* was found to be a purine base located 26 nucleotides upstream of the ATG start codon (Fig. 1B). Examination of the sequence upstream of the +1 site revealed candidate −10 and −35 hexamers with strong resemblance to the *E*. *coli* sigma-70 binding sequence, and separated by 16 base pairs. The −10 sequence matches the *E*. *coli* sigma 70 consensus sequence at four of six nucleotides, while the −35 sequence matches at five out of six nucleotides. Within the 5’ untranslated region of *ipdC* is a strong candidate ribosome binding site. This positions the center of the strong TyrR box 33 base pairs upstream from the center of the *ipdC* −35 sequence, roughly three turns of the DNA helix.

5’-RACE identified a transcription start site for the *akr* gene 19 base pairs downstream of the proposed ATG start codon at an adenine residue. This was considered to be an artifact based on the following evidence: (i) the *akr* coding sequence is conserved among closely related bacteria with annotated genomes, including the start codon, and (ii) the identified adenine was located at the end of five contiguous adenines, and reverse transcriptase is known to pause or prematurely terminate activity when it encounters a poly-adenine tract [34]. The genome wide transcription start sites of the closely-related enterobacterium *Salmonella enterica* sv. *typhimurium* SL1344 were identified by RNA sequencing, including those for the homologous *ipdC* and *akr* genes [35]. Alignment of the *S*. *enterica* SL1344 and *E*. *cloacae* UW5 *akr*-*ipdC* regions suggested that the *E*. *cloacae* UW5 *akr* transcription start site is a cytosine nucleotide 25 base pairs upstream of the *akr* start codon (Fig. 1B). The *S*. *enterica ipdC* transcription start site identified by Kroger *et al*. (2012) is consistent with the *E*. *cloacae* UW5 *ipdC* transcription start site identified in this study. Visual inspection of the sequence upstream of the predicted *E*. *cloacae* UW5 *akr* transcription start site revealed a possible −10 element that matches four out of six nucleotides of the sigma-70 consensus sequence [36]. This positions the predicted TyrR weak box to overlap the −10 element which supports the proposal that TyrR functions as a repressor of *akr*. No definitive −35 hexamer was identified upstream of the −10, although the lack of a −35 element does not preclude RNA polymerase interaction [37]. A candidate ribosome binding site sequence was found seven base pairs upstream of the ATG within the 5’-UTR of *akr*.

### ***ipdC*** is positively regulated by TyrR and the aromatic amino acids

**Fig 2:**
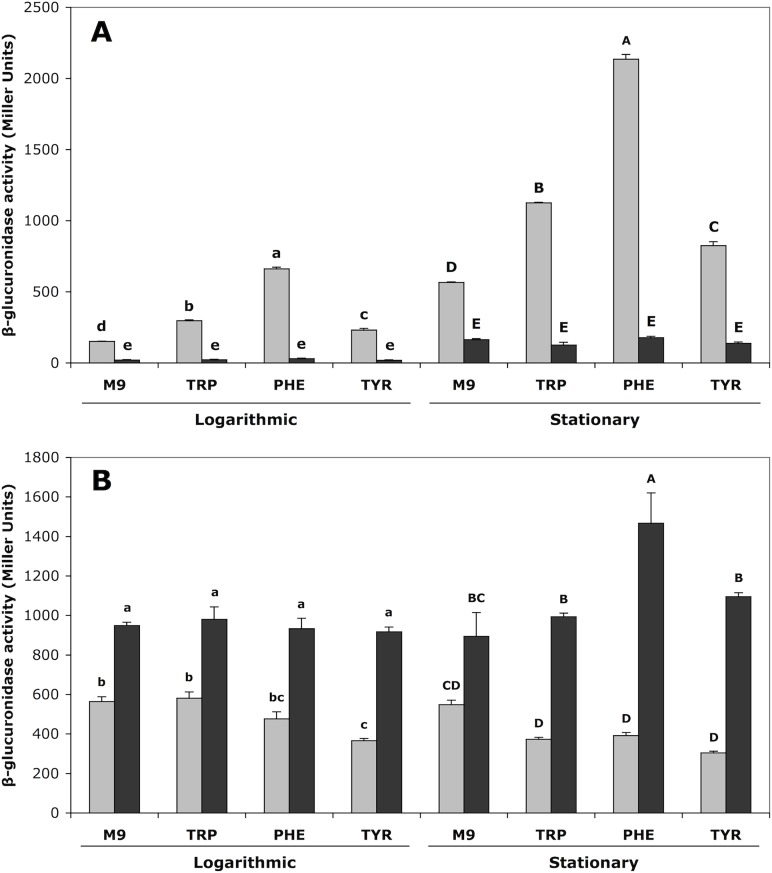
The expression of *akr* and *ipdC* is modulated by TyrR. Expression from (A) *ipdC* and (B) *akr* in the presence or absence of aromatic amino acids in wild-type *E*. *cloacae* UW5 (grey) and *tyrR* null mutant *E*. *cloacae* J35 (black). Cells were assayed for β-glucuronidase activity in both logarithmic and stationary phases of growth. Error bars represent the standard error of the means of three independent replicates. Statistically significant differences of *p* < 0.05 are indicated by lowercase letters in logarithmic phase, and uppercase letters in stationary phase.

To study the regulatory effects of TyrR on the *akr* and *ipdC* genes, *uidA* transcriptional fusions were generated using a derivative of the low copy number plasmid pBBR1MCS-2 [30] which lacks the *lacZ* promoter. Consistent with previous results [25], when wild-type *E*. *cloacae* UW5 cells containing the *ipdC*::*uidA* fusions were grown and assayed for β-glucuronidase activity in stationary phase, expression from P*ipdC* was 3.5-fold greater than that in the *tyrR* null mutant *E*. *cloacae* J35 strain which lacks a functional TyrR (Fig. 2A). In the wild-type background, expression from *ipdC* increased significantly in response to each of the aromatic amino acids, relative to that in M9 minimal medium (Fig. 2A). Phenylalanine and tryptophan had the strongest effects, increasing *ipdC* expression four-fold and two-fold, respectively, above M9 minimal media alone in stationary phase. Tyrosine also induced expression 1.5-fold above that in minimal media, however this was less then that previously observed [25]. Expression of *ipdC* was on average 3.6-fold greater in stationary phase relative to logarithmic phase. This is consistent with the observation that IAA, the product of the reaction catalyzed by IPDC, is not detectable in the culture media of *E*. *cloacae* UW5 until stationary phase [25, 29].

### ***akr*** is negatively regulated by TyrR

In contrast to the positive regulatory effects of TyrR on the *ipdC* promoter, *akr* expression was negatively regulated by TyrR (Fig. 2B). When expression from *akr* was measured in the *tyrR* null mutant *E*. *cloacae* J35, a 1.6-fold increase was observed (in both log and stationary phases) compared to expression levels in wild-type *E*. *cloacae* UW5, indicating that TyrR normally represses *akr*. Inclusion of the aromatic amino acids tryptophan and phenylalanine did not result in significant down-regulation of *akr*, however, the inclusion of tyrosine in the medium resulted in a 1.5-fold decrease in *akr* expression relative to M9 medium alone during log phase. Transcription from the *akr* promoter remained at relatively constant levels during both logarithmic and stationary phases of growth when assayed in wild-type *E*. *cloacae* UW5.

### ***ipdC*** activation requires the strong TyrR binding site

**Fig 3:**
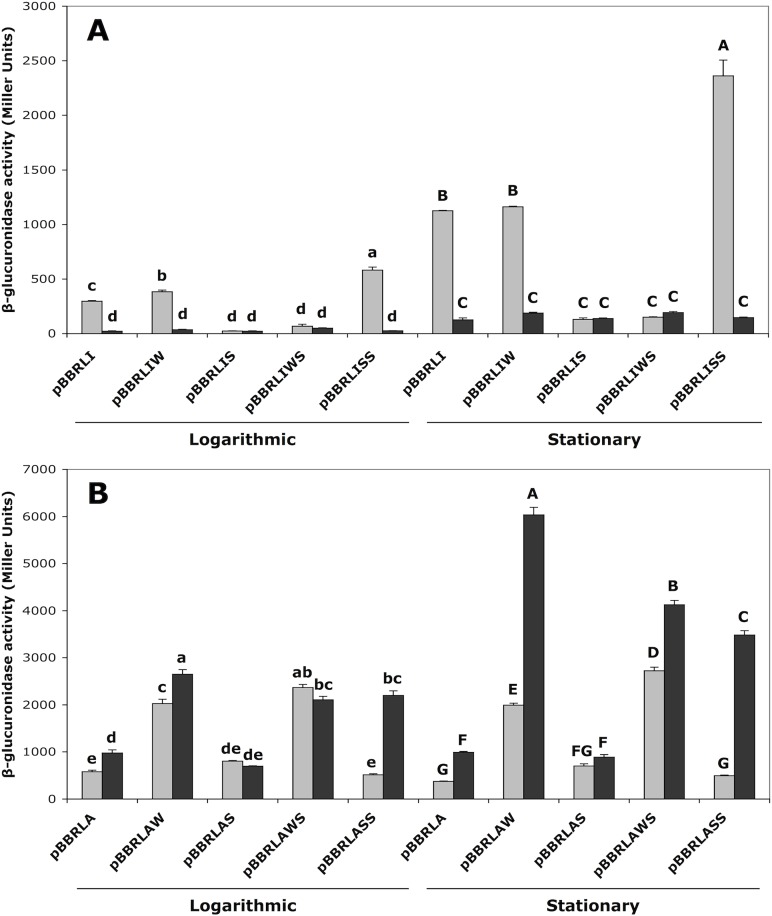
Role of the TyrR boxes in *akr-ipdC* gene regulation. Expression from (A) *ipdC* and (B) *akr* promoter mutants in wild-type *E*. *cloacae* UW5 (grey) and *tyrR* null mutant *E*. *cloacae* J35 (black) in tryptophan-supplemented M9 minimal media. Cells were assayed for β-glucuronidase activity in both logarithmic and stationary phases of growth. Error bars represent the standard error of means of three independent replicates. Statistically significant differences of *p* < 0.05 are indicated by lowercase letters in logarithmic phase, and uppercase letters in stationary phase.

To test the involvement of the two predicted binding sites in TyrR mediated activation of *ipdC*, four promoter variants were constructed via SLIM-PCR mutagenesis (Fig. 1C). These mutations were generated to abolish TyrR binding to the weak and/or strong boxes by changing nucleotides known to be essential for TyrR DNA binding in *E*. *coli* [1]. The center of the strong TyrR box is located 33 base pairs upstream of the center of the predicted −35 element of the *ipdC* promoter, positioning the two sites three turns of the DNA helix apart and on the same face of the DNA. This would allow TyrR to act as a strong type I transcriptional activator in a manner consistent with other genes that are positively regulated by TyrR, such as *tyrP* [38]. Mutation of the strong box (strains carrying pBBRLIS) resulted in the reduction of *ipdC* expression to a level similar to that in the *tyrR* null mutant *E*. *cloacae* J35 (Fig. 3A). Similar results were observed from the double weak and strong box mutant promoter (pBBRLIWS). In both logarithmic and stationary phase, inactivation of the weak box alone (pBBRLIW) was found to have little effect on expression of *ipdC* (Fig. 3A).

To examine the effect of TyrR binding at the weak box on the *ipdC* promoter, the weak box spacing was adjusted such that each arm of the palindromic TyrR box motif was separated by the ideal six nucleotides (corresponding to the same spacer nucleotides as in the adjacent strong box). The high identity of the weak box palindromic arms to the TyrR consensus sequence, coupled with the optimal spacing would ensure that TyrR is bound with high affinity to both sites [1]. The resulting double strong box promoter (pBBRLISS) increased transcriptional activity 2.1-fold over levels of the wild-type promoter (pBBRLI) (Fig. 3A). Similar expression trends were observed in the presence and absence of aromatic amino acids, although this effect was enhanced in the presence of aromatic amino acids, especially phenylalanine, as expected for a TyrR mediated response (S1 Fig.).

### Repression of ***akr*** is facilitated by the weak TyrR binding site occluding the promoter

Based on the predicted transcription start site for *akr*, the weak TyrR box is situated to overlap the predicted −10 sequence of the *akr* promoter, suggesting that binding of TyrR to the weak box competes with RNA polymerase for access to the promoter. Mutation of the weak box (pBBRLAW) resulted in a 3.5- and 5.3-fold higher level of expression from the *akr* promoter compared to the wild-type promoter (pBBRLA) in logarithmic and stationary phases, respectively (Fig. 3B). The effect of the weak box mutation was greater in the *tyrR* null mutant *E*. *cloacae* J35, particularly in stationary phase, which showed a 16-fold increase in *akr* expression over the wild-type UW5 strain. Inactivation of the strong box alone (pBBRLAS) had no effect on *akr* expression in either growth phase suggesting that the observed repression is mediated by the weak box (Fig. 3B). When both TyrR boxes were mutated (pBBRLAWS), transcription from the *akr* promoter increased 7.3- and 11-fold over levels from the wild-type promoter in both wild-type *E*. *cloacae* UW5 and the *tyrR* null mutant *E*. *cloacae* J35, respectively, in stationary phase. Conversion of the weak box to a strong box (pBBRLASS) yielded the same level of repression as from the wild-type promoter in wild-type *E*. *cloacae* UW5; however, in the *tyrR* null mutant *E*. *cloacae* J35, transcription increased 3.8- and 9.3-fold in log and stationary phases, respectively. The effect of the TyrR box mutations on *akr* expression was similar in M9 medium with and without aromatic amino acid supplements (S2 Fig.). These data indicate that both the weak box and TyrR are important for *akr* repression, but that TyrR interaction at the weak box does not sufficiently explain the observed repression.

### TyrR binds to two sites in the ***akr-ipdC*** intergenic region

**Fig 4:**
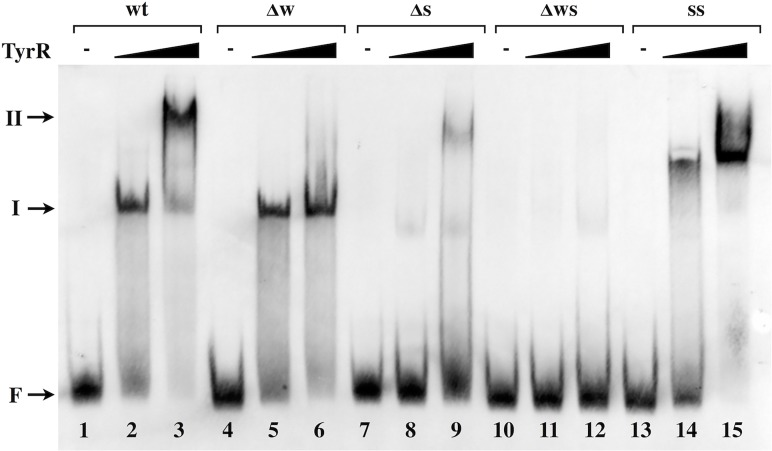
Binding of TyrR to the TyrR boxes within the *akr-ipdC* intergenic region. DIG-labeled DNA probes correspond to the *akr-ipdC* intergenic sequence of the template plasmid from which they were generated, wild-type sequence (Lanes 1–3); weak box mutant (Lanes 4–6); strong box mutant (Lanes 7–9); double weak and strong box mutant (Lanes 10–12) and double strong boxes (lanes 13–15). DNA probes were incubated with either no TyrR (Lanes 1, 4, 7, 10 and 13) or increasing concentrations of TyrR (87 nM: Lanes 2, 5, 8, 11 and 14; 877 nM: Lanes 3, 6, 9, 12 and 15). Arrows indicate the positions of free DNA (F) and the two resolved TyrR-DNA complexes (I, II).

The ability of TyrR to bind to specific sites within the intergenic region of *akr-ipdC* was demonstrated using purified His_6_-TyrR protein. When incubated with purified His_6_-TyrR, a DIG-labeled DNA fragment encompassing the wild-type *akr-ipdC* intergenic region migrated to a position consistent with a higher molecular weight complex on a native-PAGE gel (complex I), compared to the DNA fragment alone (Fig. 4, lanes 1, 2). Migration of the DNA fragment was His_6_-TyrR concentration dependent, with levels of His_6_-TyrR greater than 87 nM inducing a second DNA shift to complex II (Fig. 4, lane 3, and S3 Fig.). These results suggested that TyrR binds to two sites in the *akr-ipdC* intergenic region. Pre-incubation of His_6_-TyrR with a 10-fold molar excess of unlabeled DNA probe abolished the band at the complex II location, however complex I was still apparent in the gel (S3 Fig., rightmost lane). There was no apparent increase or decrease in affinity for DNA binding by His_6_-TyrR when EMSAs were repeated in the presence of aromatic amino acids (S3 Fig.).

To determine if His_6_-TyrR binds to the *akr-ipdC* intergenic region at the two predicted TyrR boxes, EMSAs were performed using the mutated promoter sequences generated by SLIM-PCR as DNA binding templates for His_6_-TyrR. The results show that when the weak box was mutated, His_6_-TyrR still bound the DNA to yield complex I, however complex II was abolished (Fig. 4, lanes 5, 6). Mutations in the strong box resulted in the loss of intense bands representing both complexes, although the weak bands may indicate some low affinity interactions with the intact weak box at higher His_6_-TyrR concentrations (Fig. 4, lanes 8, 9). All interactions were abolished with fragments carrying mutations in both TyrR boxes (Fig. 4, lanes 11, 12). When the DNA fragment with the double strong box was used as a template for EMSAs, all labeled DNA migrated to complex II, even at the lowest concentration of His_6_-TyrR (Fig. 4, Lanes 14, 15). This indicates that the increased spacing between the palindromic arms of the weak box increased the affinity of TyrR for the weak box and supports strong protein-protein interactions between TyrR dimers bound at both locations on the DNA. The results shown in Fig. 4 demonstrate that TyrR interacts with the two predicted TyrR binding sites within the *akr-ipdC* intergenic region. While interactions with the *akr* proximal box are weaker and require TyrR to be bound to the adjacent strong box *in vitro*, this is not the case *in vivo* where the strong box has no discernible effect on *akr* expression. In the latter case, additional regulatory factors may be acting at the promoter.

## Discussion

In this report we detail the transcriptional control of two adjacent, divergently transcribed genes, *akr* and *ipdC*, by the transcription factor TyrR, elucidated by promoter fusion expression assays and EMSAs to assess protein-DNA interactions. Expression of *akr* and *ipdC* was controlled by TyrR interaction with a weak and strong TyrR box, respectively, located within the intergenic region. Direct protein-DNA interactions between TyrR and the strong and weak boxes were shown *in vitro* using purified TyrR protein and DNA fragments containing the *akr-ipdC* intergenic region; the interactions were abolished when point mutations were introduced in either or both TyrR boxes. These interactions were strengthened when the sequence of the weak box was altered to match that of a strong box as demonstrated by the shift of the DNA fragment to a higher molecular weight complex when incubated with lower concentrations of TyrR and resolved by EMSA, and by the increased expression from *ipdC* reporter fusions.

Expression of *ipdC* is dependent on TyrR for activation; in the *tyrR* null mutant *E*. *cloacae* J35, expression of the P*ipdC*::*uidA* fusion was abolished (this study; [25]), and production of IAA is also reduced. *E*. *cloacae* indolepyruvate decarboxylase, encoded by *ipdC*, catalyzes the production of IAA [25, 39]. In plants, IAA is an important growth hormone, controlling many aspects of growth and morphogenesis such as gravitropism, cell elongation and tissue differentiation [40]. *E*. *cloacae* UW5 inhabits the rhizosphere of plants, where it may produce high levels of IAA, and has been shown to positively influence root development [29]. Plant growth-promoting rhizobacteria have been applied in field studies and commercial agriculture to improve crop yields, although results are often inconsistent or improvements only marginal [41]. Identifying the mechanisms underpinning regulation of IAA production in plant growth-promoting rhizobacteria will aid in their application as agricultural phytostimulants, especially considering that IAA is also a virulence factor produced by some plant pathogens and levels of this hormone may be an important factor in plant interactions (recently reviewed by [26]).

Inclusion of any of the aromatic amino acids in the growth media significantly increased *ipdC* expression in a TyrR dependent manner, with phenylalanine as the strongest inducer. The center of the strong TyrR box is located 33 base pairs upstream from the center of the predicted *ipdC* −35 element. This would position a TyrR dimer bound at the strong box on the same face of the DNA as RNA polymerase bound to the *ipdC* promoter, and in an optimal location for interactions with the RNA polymerase α-CTD [1, 38]. It is likely that the strong induction of *ipdC* is due to activation by TyrR complexed with aromatic amino acids, which increases the affinity of TyrR for the DNA and leads to strong activation in promoters with similarly positioned TyrR boxes [21, 42]. The promoter of the *mtr* gene in *E*. *coli* has a similarly orientated strong TyrR box centered 42 base pairs upstream of the −35 element. In the presence of phenylalanine, a TyrR dimer bound to this strong box interacts with the RNA polymerase α-CTD enhancing expression 2.8-fold [17, 21]. A comparison of the TyrR activation of the *E*. *coli mtr* promoter and the *E*. *cloacae* UW5 *ipdC* promoter reveals very similar trends. The *mtr* gene is activated 2-fold (tryptophan), 2.8-fold (phenylalanine) and 1.7-fold (tyrosine); while *ipdC* is activated 2-fold (tryptophan), 3.7-fold (phenylalanine) and 1.4-fold (tyrosine) [20].

**Fig 5:**
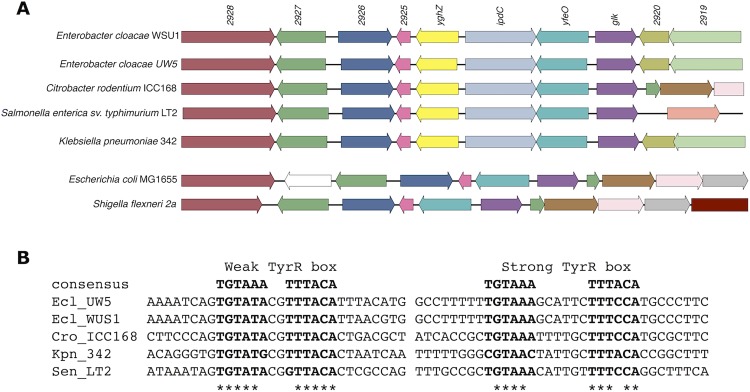
Conservation of the *akr-ipdC* genes and the two TyrR boxes across closely related bacteria. (A) Genomic context of the *akr-ipdC* genes in closely related *Enterobacteriaceae*, Homologous genes are indicated by the same colour, white indicates non-homologous genes. (B) Species with an *akr-ipdC* gene pair retain the weak and strong TyrR boxes. The second palindromic arm of the *E*. *cloacae* UW5 weak TyrR box is not conserved across species.

The newest member of the TyrR regulon, *akr*, identified in this study, was shown to be down-regulated by TyrR. Little is known regarding the function of the protein encoded by the *akr* gene. From the amino acid sequence it is predicted to be a member of the aldo-keto reductase (AKR) superfamily of proteins, a diverse group of enzymes found in all domains of life that share a common (α/β)_8_ barrel fold and utilize the nicotinamide adenine dinucleotide (NAD(P)H) cofactor to reduce carbonyl compounds [43, 44]. AKRs are further divided into families based on amino acid sequence identity (at least 40% identity for family designation). Phylogenetic analysis of the *E*. *cloacae* UW5 AKR groups it with *E*. *coli* YghZ (data not shown), which is homologous to the AKR6 family that comprises the β-subunit of voltage gated potassium channels [45]. The *yghZ* gene encodes a methylglyoxal reductase, which has been shown to confer protection against the toxic electrophile methylglyoxal by reducing it to acetol [46], and has been designated ARK14A1 based on amino acid identity. Although the function of the *E*. *cloacae* UW5 AKR is unknown, the sequence and organization of the open reading frame is conserved among closely related bacteria that possess the *ipdC* gene: when *ipdC* is present in a sequenced enterobacterial genome, it is found adjacent to, and divergently transcribed from, a homologous *akr (* Fig. 5A). Both the strong and weak TyrR bindings sites are also conserved in these genomes (Fig. 5B). Thus, the common regulation of these two genes by TyrR and their evolutionarily conserved pairing suggest a functional relationship between AKR and IAA biosynthesis.

Down-regulation of *akr* is dependent on the weak TyrR box which overlaps the predicted −10 element of the *akr* promoter; however, mutations in the strong TyrR box upstream of the *akr* promoter had no effect on expression. The observation that expression from the *akr* mutant weak box promoter was higher than that from the wild-type promoter in the *tyrR* null mutant *E*. *cloacae* J35, when the expectation was that they would be similar, may indicate that the nucleotide changes in the weak box increased the strength of the *akr* promoter. Alternatively, the increase in expression of the mutant weak box promoter may indicate the involvement of a second regulatory protein. This is supported by the similarly high levels of *akr* expression in the *tyrR* null mutant *E*. *cloacae* J35 containing the mutant weak or double strong box promoters. In both cases, nucleotide changes were introduced in the weak box sequence (in the case of the double strong box these would not be expected to enhance promoter strength) and may have abolished the binding site for a second regulator. In wild-type *E*. *cloacae* UW5 with a double strong box, the increased affinity of TyrR for the weak box maintained repression of *akr* at wild-type levels.

The observation that *akr* was down-regulated by TyrR, but not completely repressed may reflect the weak interactions between TyrR and the DNA binding site overlapping the −10 element. A TyrR dimer or hexamer may associate transiently with the weak box allowing RNA polymerase access to the promoter and a low level of expression. This is proposed to be the case with the *aroG* gene of *E*. *coli*, where the TyrR box overlaps the −10 element and competition between the two proteins for DNA binding results in a low but constant level of gene expression [1]. Weak interactions between TyrR and the weak box may be stabilized by protein-protein interactions between TyrR bound to both boxes. These may be dimer-dimer interactions, as in the case of *mtr* [17] or the formation of a TyrR hexamer. TyrR is thought to polymerize from a dimer to a hexamer in the presence of tyrosine while bound to a strong box sequence which acts as a nucleation point [7]. The resulting hexamer has a 2- to 5-fold increased affinity for DNA binding, allowing its interaction with adjacent, weaker binding sites [47]. TyrR may polymerize when bound to the strong box in the *akr-ipdC* intergenic region, increasing its affinity for the adjacent weak box in the presence of tyrosine. This is supported by the requirement for the wild-type strong box for strong TyrR binding to the intergenic region in EMSAs. The observation that mutations in the strong box do not affect *akr* expression in the reporter gene assays may support the involvement of a second regulatory protein that stabilizes TyrR binding to the weak box *in vivo*.

The functionality of the weak TyrR box, demonstrated by its necessary role in repression of *akr in vivo* and by its binding to TyrR *in vitro* as shown by EMSAs, was one of the most interesting findings of this study. In *E*. *coli*, TyrR binds the consensus sequence TGTAAA-N_6_-TTTACA and requires the conserved G-N_14_-C pair [1]. The atypical spacing between the weak box arms in the *akr* promoter and the double tandem 5’ arms suggests two possibilities for the spacing of the G-C pair: G-N_10_-C or G-N_16_-C. There is little information regarding protein binding to DNA sequences with atypical spacing between palindromic half-sites. The consensus binding sequence for PrrA, a global transcription factor controlling ~25% of the genome in *Rhodobacter sphaeroides* [48], comprises two inverted half-sites separated by a spacer region of variable length, ranging from 0 to 10 base pairs [49]. Site directed mutagenesis coupled with promoter fusions and EMSAs revealed that while a five base pair spacer is optimal, PrrA also interacts with sites with an 8 base pair spacer [50].

Genome wide studies of transcription factor interactions with DNA have revealed that interactions with non-canonical sites, which were previously considered artifacts, are more common then previously thought and reflect true protein-DNA interactions. Chromatin immunoprecipitation coupled with microarray analysis (ChIP-chip) performed with the LexA regulator of *E*. *coli* demonstrated its interaction *in vivo* with several promoter regions lacking a discernible LexA DNA binding consensus sequence, while these sites were bound extremely poorly when studied *in vitro* [51]. ChIP-chip performed with the nitric oxide responsive NsrR regulator of *E*. *coli* revealed that the protein can recognize and bind to two types of binding sites: one comprised of two 11 base pair inverted repeats separated by a single nucleotide and the other contains only a single 11 base pair arm, with no discernible second inverted arm [52]. While in some cases, interactions between transcription factors and non-canonical sites have no discernible effect on gene transcription [51, 53], we have shown that interaction between TyrR and the weak box results in the down-regulation of *akr* expression.

## Supporting Information

S1 Fig Expression of *ipdC* in promoter mutants by TyrR in response to aromatic amino acids. Expression from *ipdC* promoter mutants in M9 minimal medium without amino acid supplements (A) and in the presence of phenylalanine (B), or tyrosine (C) in wild-type *E*. *cloacae* UW5 (grey) and *tyrR* null mutant *E*. *cloacae* J35 (black). Cells were assayed for β-glucuronidase activity in both logarithmic and stationary phases of growth. Error bars represent the standard error of the means of three independent replicates. Statistically significant differences of *p* < 0.05 are indicated by lowercase letters in logarithmic phase, and uppercase letters in stationary phase.
(TIF)

S2 Fig Expression of *akr* promoter mutants by TyrR in response to aromatic amino acids. Expression from *akr* promoter mutants in M9 minimal medium without amino acid supplements (A) and in the presence of phenylalanine (B), or tyrosine (C) in wild-type *E*. *cloacae* UW5 (grey) and *tyrR* null mutant *E*. *cloacae* J35 (black). Cells were assayed for β-glucuronidase activity in both logarithmic and stationary phases of growth. Error bars represent the standard error of the means of three independent replicates. Statistically significant differences of *p* < 0.05 are indicated by lowercase letters in logarithmic phase, and uppercase letters in stationary phase.
(TIF)

S3 Fig Binding of TyrR to the *akr-ipdC* intergenic region is concentration dependant. Wild-type DIG-labelled DNA probe was incubated with increasing amounts of TyrR-His_6_ (0 mM, 87 nM, 438 nM, 877 nM 1750 nM) in the absence of aromatic amino acids (A), 1 mM tryptophan (B), phenylalanine (C) or tyrosine (D). Competition assays were performed with 10-fold molar excess of unlabeled-probe over DIG-labelled probe; rightmost lane. Positions of free DNA (F) and the two resolved TyrR-His_6_-DNA complexes (I, II) are indicated.
(TIF)
