# Supplementary figures and images for: The TyrR Transcription Factor Regulates the Divergent akr-ipdC Operons of Enterobacter cloacae UW5
Source: PLoS One. 2015 Mar 26;10(3):e0121241. doi: 10.1371/journal.pone.0121241 (PMC4374768; doi:10.1371/journal.pone.0121241)

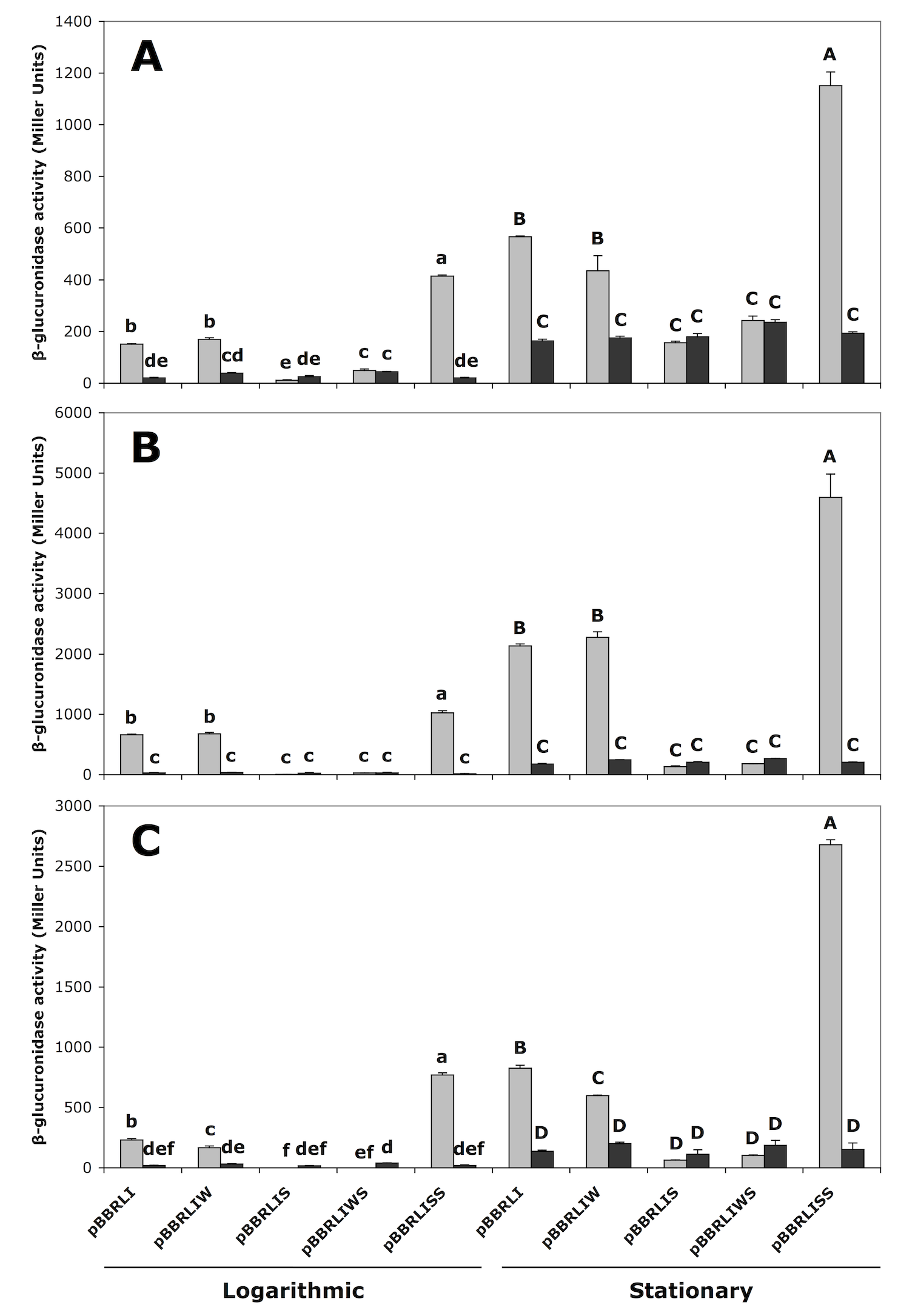

Supplement: S1 Fig — Expression from ipdC promoter mutants in M9 minimal medium without amino acid supplements (A) and in the presence of phenylalanine (B), or tyrosine (C) in wild-type E. cloacae UW5 (grey) and tyrR null mutant E. cloacae J35 (black). Cells were assayed for β-glucuronidase activity in both logarithmic and stationary phases of growth. Error bars represent the standard error of the means of three independent replicates. Statistically significant differences of p < 0.05 are indicated by lowercase letters in logarithmic phase, and uppercase letters in stationary phase. (TIF) [file pone.0121241.s001.tif]

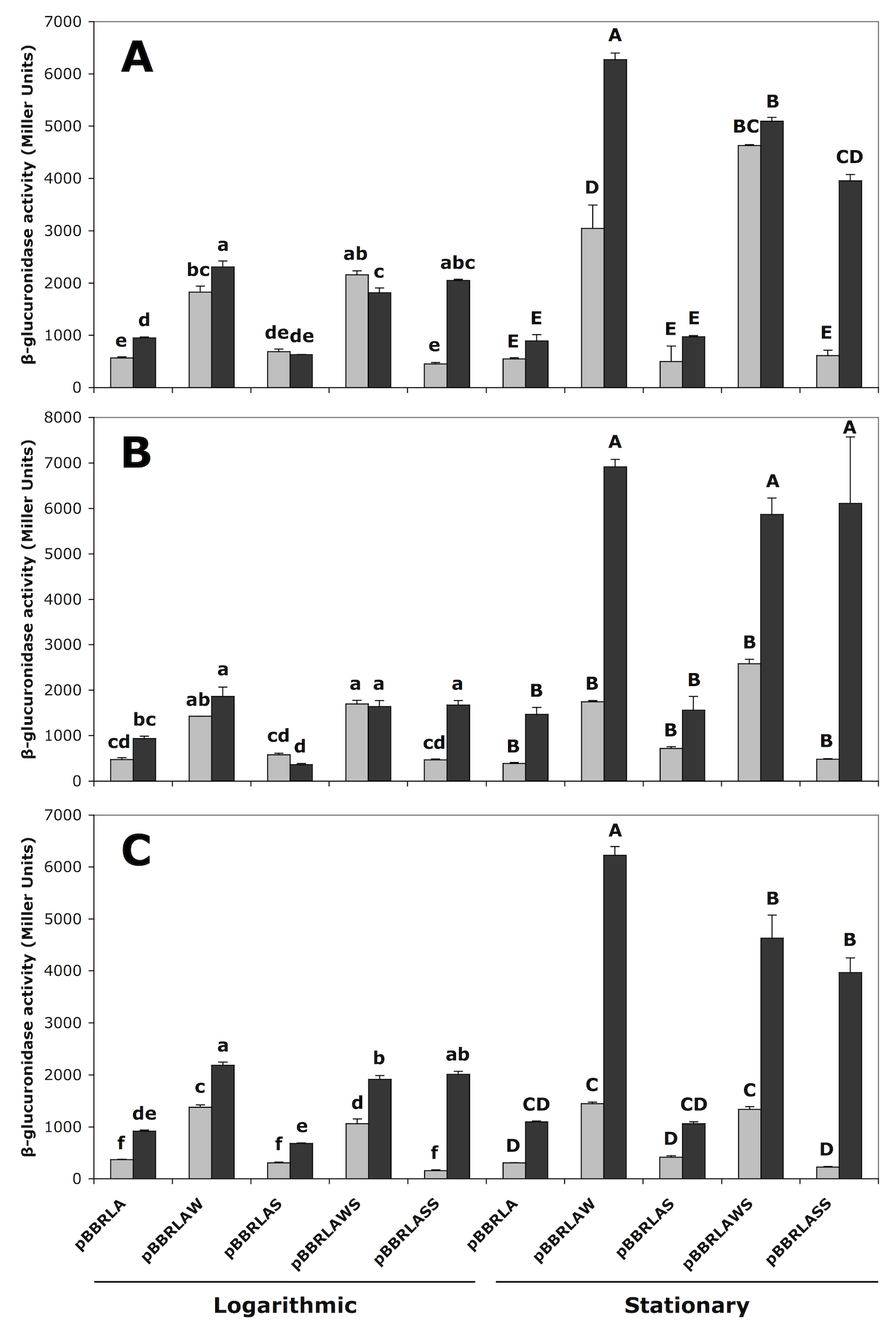

Supplement: S2 Fig — Expression from akr promoter mutants in M9 minimal medium without amino acid supplements (A) and in the presence of phenylalanine (B), or tyrosine (C) in wild-type E. cloacae UW5 (grey) and tyrR null mutant E. cloacae J35 (black). Cells were assayed for β-glucuronidase activity in both logarithmic and stationary phases of growth. Error bars represent the standard error of the means of three independent replicates. Statistically significant differences of p < 0.05 are indicated by lowercase letters in logarithmic phase, and uppercase letters in stationary phase. (TIF) [file pone.0121241.s002.tif]

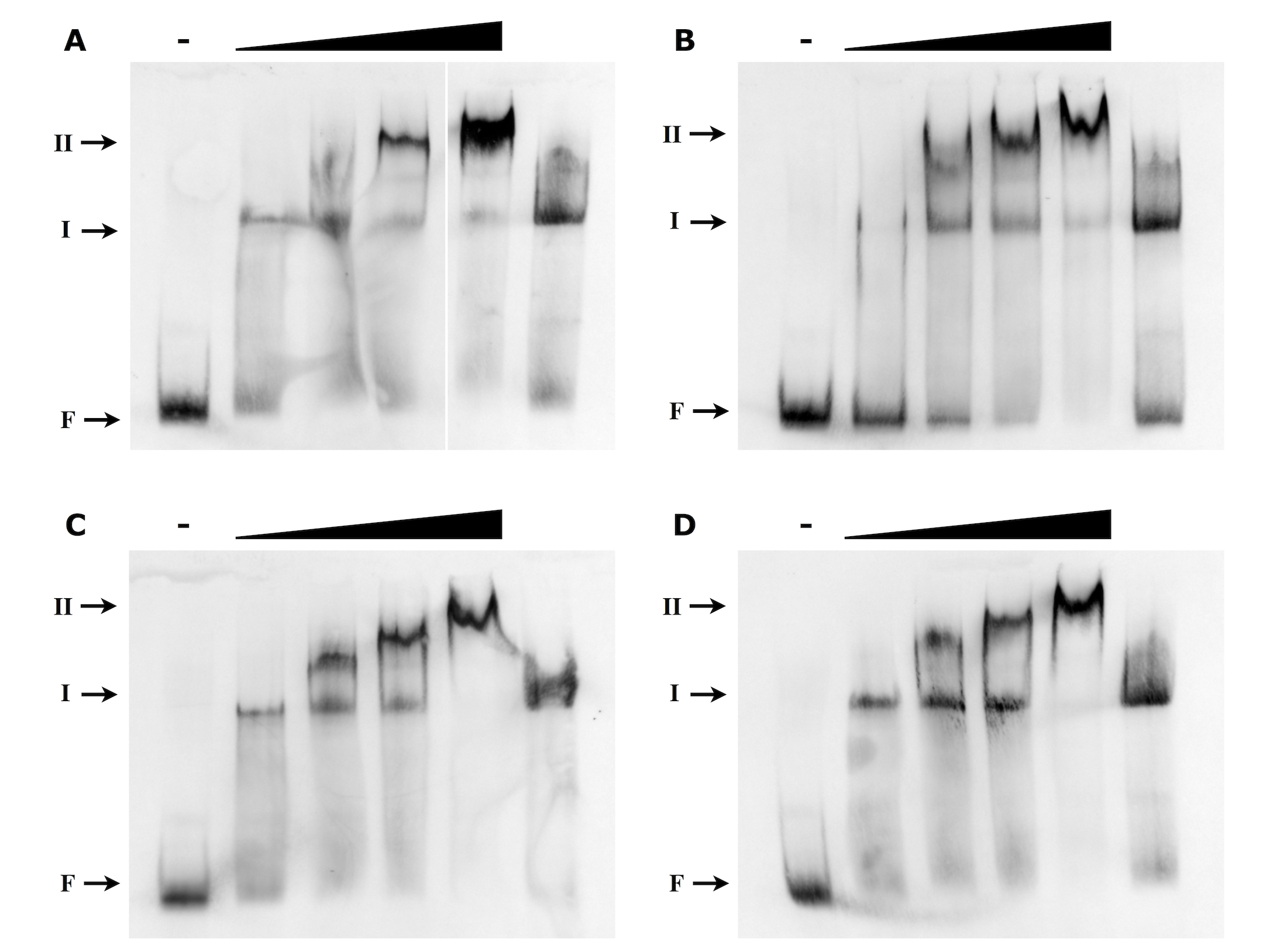

Supplement: S3 Fig — Wild-type DIG-labelled DNA probe was incubated with increasing amounts of TyrR-His6 (0 mM, 87 nM, 438 nM, 877 nM 1750 nM) in the absence of aromatic amino acids (A), 1 mM tryptophan (B), phenylalanine (C) or tyrosine (D). Competition assays were performed with 10-fold molar excess of unlabeled-probe over DIG-labelled probe; rightmost lane. Positions of free DNA (F) and the two resolved TyrR-His6-DNA complexes (I, II) are indicated. (TIF) [file pone.0121241.s003.tif]
